# Free Nursing for the Poor

**Published:** 1888-02-25

**Authors:** 


### FREE NURSING FOR THE POOR.

Is there any institution which provides nurses for the poor in very poor parishes which have no funds to pay the nurse? Or is there any institution which gives a grant for this purpose? This is a very large parish and a very poor one, and we have constantly cases where a nurse would be invaluable, and I am told that there is a society for providing district nurses, but I do not know where to apply. E. C. R.

The fatal lack of power to combine shown by women is perfectly sickening. No sooner is a Pension Fund started, than a British Nurses' Association follows suite; no sooner is The Hospital published, than "Nursing Notes" is announced; and now we are threatened with a "Nursing Record." A house divided against itself cannot stand, and in spite of the charitable efforts of the public, the nurses will soon find that if they do not all pull together no external aid can raise their profession to the honoured platform where it ought to rest. Nurses have left the trivialities of social life, and entered each on a noble career; yet petty spites and jealousies seem to revel in their midst, and cliques of all kinds to abound. Surely some greatness of spirit might be expected from these women which would lead them to put aside their individual schemes, and all unite together for mutual sympathy and support. H. M.

February 21st, 1888.

